# Case report: Case series featuring anastomotic colonic adenocarcinoma following jejunoileal bypass requiring oncologic resection and jejunoileal bypass reversal

**DOI:** 10.3389/fsurg.2023.1249441

**Published:** 2023-10-06

**Authors:** Bryan Miles, Anthony Visioni, Christopher Daigle, Robert Marley, Stephen Brandstetter

**Affiliations:** ^1^ Department of Surgery, Cleveland Clinic Akron General, Akron, OH, United States; ^2^ Department of Surgery, Kaiser Permanente Washington Health Research Institute, Seattle, WA, United States

**Keywords:** jejunoileal bypass, colon adenocarcinoma, jejunoileal bypass reversal, bariatric surgery, case series

## Abstract

Like all surgical fields, bariatric surgery has evolved immensely, so much so that previous procedures are now obsolete. For instance, the jejunoileal bypass has fallen out of favor after severe metabolic consequences resulted in prolonged morbidity and even mortality. Despite this, several patients persevered long enough to develop other pathology, such as cancer. This progression has been validated in animal models but not human patients. Nonetheless, contemporary surgeons may encounter situations where they must resect and re-establish intestinal continuity in patients with this antiquated anatomy. When faced with this scenario, the question of whether or not the previously bypassed small bowel can be safely reunited plagues the surgeon remains unanswered. Unfortunately, the literature does not effectively answer this question, even anecdotally through case reports or series. Therefore, we share our experience with three patients who developed colon cancer following jejunoileal bypass and subsequently underwent oncologic resection with simultaneous reversal of their jejunoileal bypasses.

## Introduction

**Figure 1:**
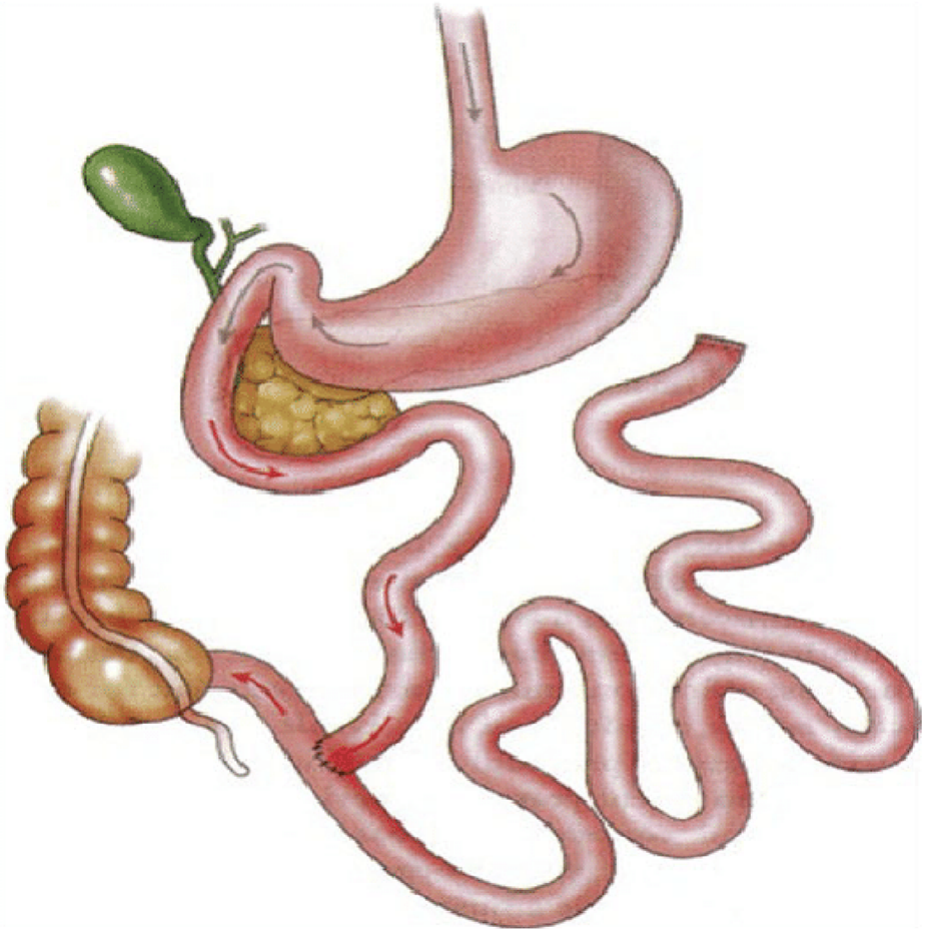
Traditional jejunoileal bypass schematic highlighting bypassed small bowel and reanastomosis near the colon. Subsequent iterations involved reanastomosis of bypassed small bowel into the colon in various locations (transverse and sigmoid colon) (4).

While previous weight loss procedures may seem historic, modern surgeons still encounter these anatomic relics. The jejunoileal bypass (JIB), first performed on a human in 1954, involved an end-to-side anastomosis between the jejunum and terminal ileum (Figure 1). Depending on the amount of bowel bypassed, the jejunum was typically transected 40 cm past the ligament of Treitz and attached 25 cm proximal to the ileocecal valve. Despite the dramatic weight loss, profound metabolic consequences, which include severe diarrhea, electrolyte abnormalities, liver failure, renal insufficiency, biliary and renal lithiasis, and bone disease, all of which require frequent hospitalizations and may even cause death, led to the abandonment of the procedure a few decades later and ultimate reversal in many patients. Unfortunately, the reversal resulted in a rapid return of previously achieved weight loss. Modification of the jejunoileal bypass by anastomosis of the bypassed segment into the colon grew out of refractory weight loss, speculated to be secondary to the reflux of enteric contents and subsequent absorption of excess calories (1–3). Other less frequently cited associations include carcinoma. These are described only in case reports, ranging from numerous sessile polyps necessitating total abdominal colectomy (4) to intussusception of the defunctionalized ileum into the sigmoid colon secondary to lymphoma (5), to squamous cell of the rectum (6), or to adenocarcinoma of the ileocecal valve (7). The unique element of the case series presented herein lies in the reversal of the jejunoileal bypass and post-operative management and the finding of adenocarcinoma at the defunctionalized small bowel transverse colon anastomosis.

## Case 1

JS is a 72-year-old gentleman who underwent JIB in the 1980s. He has no additional contributory history. Despite laxatives, he presented to the emergency department with abdominal pain, distention, nausea, vomiting, and obstipation for 3 days. A colonoscopy 1 year prior was negative for polyps. A computed tomography (CT) scan upon presentation showed a 3.4-cm stenotic segment in the mid-transverse colon. Proximal to this, the ascending colon and the transverse colon were dilated and fluid-filled, with the cecum measuring 9.5 cm. There was also a loop of small bowel anastomosed to the transverse colon just proximal to the stenosis. The patient's carcinoembryonic antigen (CEA) level was 18.2. He had a bowel movement the day following admission. The day after, he underwent a colonoscopy, which showed an anastomotic obstruction in the mid-transverse colon. There was erythematous and necrotic tissue with thick and firm mucosa. Biopsies showed adenocarcinoma. The patient was discharged 6 days following admission in stable condition, including tolerance of a diet and bowel function.

**Figure 2:**
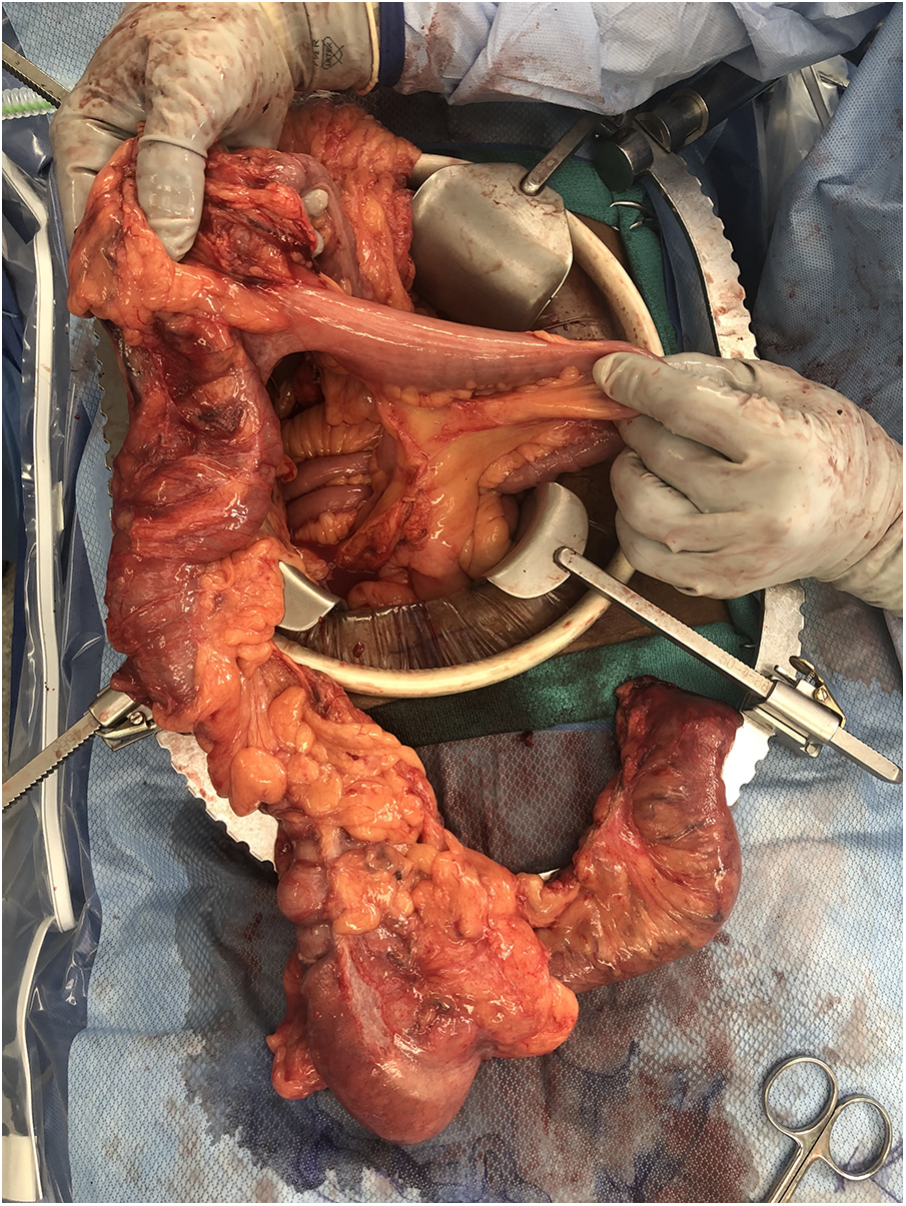
Specimen showing extended right hemicolectomy and portion of bypassed small bowel inserting into transverse colon.

**Figure 3:**
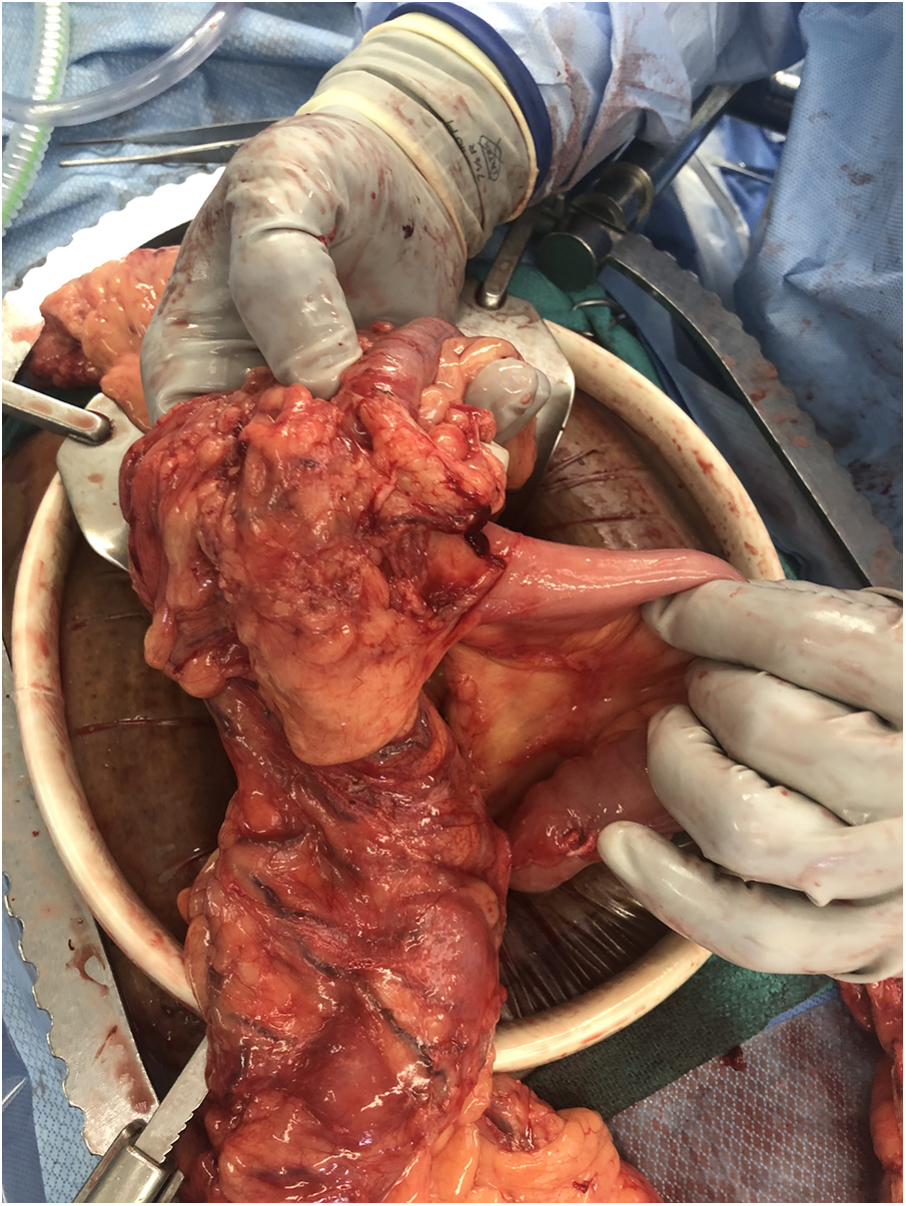
Closer view of mass at junction of bypassed small bowel and transverse colon.

Outpatient screening CT chest was negative for metastatic disease. Shortly after that, he was taken electively to the operating room for an extended right hemicolectomy with ileocolic anastomosis and reversal of JIB. Intraoperatively, the patient's anatomy revealed an end-to-end JIB, with 80 cm of bowel in continuity from the ligament of Treitz to the colon and the out-of-circuit small bowel anastomosed to the mid-transverse colon, in the end-to-side fashion, just proximal to the obstructing malignancy. The extended right hemicolectomy removed the malignancy and terminal ileum distal to the end-to-end anastomosis, which was roughly 20 cm from the ileocecal valve (Figures 2, 3). Next, a side-to-side small bowel anastomosis was performed from the terminal ileum that remained to the blind loop of the previously out-of-circuit bowel. During this process, a small portion of the small bowel was removed following trauma from extensive lysis of adhesions. At last, the portion of the small bowel previously anastomosed to the transverse colon was connected to the colon that remained after oncologic resection in an end-to-side manner.

Postoperatively, the patient was started on total parenteral nutrition (TPN) via a peripherally inserted central catheter (PICC). A CT scan on post op day (POD) 7, given no bowel function, showed diffuse ileus and slow passage of oral contrast. Flatus on POD 9 prompted a clear liquid diet, advanced to a gastrointestinal (GI) soft diet the following day with bowel movements. On POD 12, he was discharged in stable condition without TPN. Six weeks later, he was seen in the outpatient setting, with only complaint of mild constipation. He was instructed to follow up in 4 months for CEA, colonoscopy, and CT. Unfortunately, he was lost to follow-up.

## Case 2

NW is an 81-year-old woman who underwent JIB in the 1970s. She has no additional contributory medical history. She was initially admitted to the medical service for pneumonia while experiencing anemia. This prompted a workup for a GI bleed. Esophagogastroduodenoscopy (EGD) showed a 10-cm segment of salmon-colored mucosa. Biopsy was significant for Barret's. A colonoscopy the following day revealed a transverse colon mass, with the biopsy containing adenocarcinoma. At this point, general surgery was consulted. A chest, abdomen, and pelvis CT scan highlighted bilateral pulmonary embolisms and a concerning liver lesion. The liver lesion was further characterized via magnetic resonance imaging (MRI), which was significant for a 2.6-cm segment 6 mass. She subsequently began FOLFOX chemotherapy. Following four cycles, a repeat MRI scan showed stability of the area. Repeat CT chest showed a few stable lung nodules under 5 mm. a few weeks later, an abdomen and pelvis CT scan, prompted by clinical concerns of obstruction in the clinic, showed nothing acute. However, a short interval repeat CT scan showed a small bowel obstruction with a transition zone in the right lower quadrant. She was therefore admitted. Unfortunately, she was unable to advance her diet and required supplementation with TPN.

She underwent an urgent open extended right hemicolectomy, intraoperative liver ultrasound, and small bowel anastomosis secondary to the reversal of JIB. Biopsy of the liver lesion was not possible given its posterior location, but intraoperative communication with interventional radiology (IR) confirmed that a percutaneous biopsy would instead be achievable. Intraoperative findings were significant for about 200 cm of small bowel out of circuit with the distal end anastomosed to the transverse colon in an end-to-side hand-sewn fashion, which was the site of the colon cancer. The specimen included 5 cm of large bowel distal to the colon cancer, 5 cm of the distal defunctionalized small bowel limb, and just proximal to end-to-end anastomosis of the in-continuity small bowel. Reconnection of the bowel required the blind end of the small bowel in discontinuity to be anastomosed to the distal jejunum via stapled isoperistalic side-to-side anastomosis, followed by the distal end of the small bowel previously in discontinuity being anastomosed to the colon via stapled isoperistaltic side-to-side anastomosis. Pathology revealed moderately differentiated adenocarcinoma. Of 18 lymph nodes, none were positive. The final pathologic staging was T3N0M1.

By POD 8, she was tolerating a GI soft diet. She was discharged on POD 12. The patient was readmitted a few weeks later for sepsis and midline drainage, which necessitated brief admission to the ICU, vasopressors, and an infectious workup. Since this sepsis was considered secondary to her existing line, this was removed. The remainder of this hospitalization was uncomplicated, with her being discharged without further antibiotics. She presented a few months later secondary to failure to thrive, given poor PO intake. Therefore, EGD and percutaneous gastrostomy were performed without complications. An abdomen and pelvis CT scan revealed an acute PE, which was anticoagulated, and a 4-cm right hepatic lobe lesion, increased from 2 cm from previous imaging. She was ultimately discharged in stable condition on tube feeds. MRI 6 months later was significant for a 6.3-cm heterogenous lesion in the posterior right hepatic lobe and possible satellite lesions. Y 90 embolization was performed that month. A chest, abdomen, and pelvis CT scan a few months later showed disease progression, specifically numerous new bilateral pulmonary and hepatic metastases, as well as retroperitoneal and pelvic lymphadenopathy. Given the previous side effects of chemotherapy, the patient opted against further systemic therapy. She also declined hospice care. She ultimately passed away.

## Case 3

SM is a 72-year-old woman with a relevant medical history of a JIB 45 years ago and s/p left hemicolectomy in 2013 for Lynch syndrome manifesting as adenocarcinoma. A colonoscopy and associated biopsy in 2021 diagnosed her with recurrent cancer at her colorectal anastomosis. Following a negative staging CT scan, she was taken to the operating room for a JIB reversal, total abdominal colectomy, total abdominal hysterectomy (TAH), bilateral salpingo-oophorectomy (BSO), and multiple incisional hernia repair. The JIB reversal required anastomosing the previously out-of-circuit small bowel, attached to the transverse colon, proximally in the side-to-side fashion to the jejunum remaining in circuit and distally, also in the side-to-side fashion, to the rectum. The TAH and BSO were secondary to her history of Lynch syndrome. The closure repaired the incisional hernias primarily.

Postoperatively, she tolerated a clear liquid diet on POD 5, a full liquid diet on POD 6, and ultimately a GI soft diet on POD 7. She was discharged in stable condition on POD 8. She was seen in the outpatient setting without any complaints. Following readmission 1 month postoperatively for abdominal pain, a CT scan was significant for an abdominal wall fluid collection, which was managed with an IR drain. Persistent nausea prompted a repeat CT scan a week later, which showed pneumoperitoneum and a large air-containing fluid collection with concern for associated contrast material. This prompted a return to the operating room for exploratory laparotomy. Intraoperative findings included a distal small bowel perforation, not at either prior anastomosis. This area was resected, she was left in discontinuity, and a temporary wound vacuum was placed. A day later, she returned to the operating room for an end ileostomy and mucous fistula, the latter of which was created with the small portion of distal small bowel remaining. Tube feeds were started later in the day, with extubation the following day. Four days later, she was transferred to the regular nursing floor with speech-approving thin liquids. She was discharged to a skilled nursing facility, tolerating a regular diet then.

She was readmitted a week later secondary to altered mental status, decreased left-sided movement, and left-sided neglect. The stroke team workup included an MRI that showed acute small bilateral parietal infarcts. Sepsis workup given altered mental status was significant for persistent fluid collections in both the abdomen and abdominal wall; however, all were smaller than previous. The intra-abdominal collection was managed with an IR drain. Her mentation and inability to protect her airway required intubation; however, she was extubated shortly after that. Repeat CT the following day showed an obvious bowel leak with contrast extending into a large encapsulated air and fluid collection in the right lower quadrant, near the IR drain, overall decreased in size, pneumoperitoneum, and an enterocutaneous fistula. She was made nothing by mouth (NPO), a PICC was ordered for TPN, and another large bore drain was placed by IR. Ongoing dark ostomy output and anemia prompted workup by GI, including negative esophagogastroduodenoscopy, ileoscopy, and computed tomography angiography (CTA). CTA findings led to an additional IR drain into the left side, which ultimately became enteric. Finally, push enteroscopy showed a possible vascular malformation, treated by argon plasma coagulation. The midline enterocutaneous fistula appearance turned blue/green with a sweet odor, characteristic of a *Pseudomonas* infection. Cultures reflected this. A repeat CT scan and a left-sided abscessogram showed the known encapsulated fluid collection, with the known leak appearing smaller. The left-sided IR drain was upsized, while one of the right-sided drains was removed following another abscessogram. The patient was coded with an ultimate return of spontaneous circulation following advanced cardiovascular life support. Further workup was significant for a PE, for which a heparin drip was started. Bilateral pneumothoraces, likely related to the chest compressions, required chest tubes. Not long after, the family made the decision for a palliative extubation. Shortly after that, the patient passed away.

## Discussion

The pathogenesis of colonic carcinogenesis following JIB has been outlined in animal, specifically rat, models following exposure to carcinogens. One study validates the anecdotally experienced hyperplasia of the bowel remaining in the circuit, with JIB increasing the colorectal length by 17% and weight by 29% and crypt depth in the middle and distal thirds by 33% and 25%, respectively. There was also a doubling of cell proliferation in the middle third and a tripling in the distal third. Ultimately, the large bowel tumor burden was significantly increased in the bypassed rats at 30 weeks, but only 28% of these tumors were malignant. Interestingly, their data for increased bypassing of the small intestine, up to 99%, indicated no significant enhancement of large bowel tumor yields, suggesting weight reduction to be protective against colorectal neoplasia (8). Meanwhile, other similarly conducted rat models have shown no significant increase in tumor burden (9). More recently, in human cells *in vivo*, bile acids have been shown to induce carcinogenesis in colorectal cells (10). However, the few studies with human participants do not replicate these findings. One surveilled 88 patients during the years 1969–1977; however, only 30 agreed to colonoscopy. Colonoscopy performance ranged from 11 to 17 years, with a mean of 13.9 years postprocedure. Biopsies from the entirety of the colon were negative for colorectal cancer in all patients (11). Another study utilizing humans, in which biopsies were performed on 38 patients 10–13 years following JIB, saw no dysplasia or premalignant change in 371 samples (12). With respect to this case series, its strength lies in the reversal of previous short gut syndrome in its patients, the outcomes of which are meticulously documented here, while achieving a satisfactory oncologic outcome. The weakness of this approach is the potential for greater morbidity given the increasing complexity of the operation, owing to greater dissection and more anastomoses. Ironically, though, the bowel leak in case 3 did not involve an anastomosis. Unfortunately, the present literature does not detail operative findings and interventions to the detail of this series, preventing a critical comparison. In short, while a JIB has appropriately grown out of favor, these patients still present with malignant conditions. The relationship of this to their procedure has some degree of evidence, but more so in animal models. Also unanswered is what procedure to offer these patients, namely, if their previously out-of-circuit, atonic bowel should be reanastomosed. While limited, the above three cases aim to shed some light on the potential outcomes of doing just this.

## Ethical statement

Our institution’s institutional review board did not require patient consent for publication.

## Data Availability

The original contributions presented in the study are included in the article, further inquiries can be directed to the corresponding author.
